# Minimum-Cost Synthetic Genome Planning: An Algorithmic Framework

**DOI:** 10.34133/csbj.0128

**Published:** 2026-07-03

**Authors:** Michail Patsakis, Alexandros Margaris, Ioannis Mouratidis, Ilias Georgakopoulos-Soares

**Affiliations:** ^1^ Division of Pharmacology and Toxicology, College of Pharmacy, The University of Texas at Austin, Dell Pediatric Research Institute, Austin, TX, USA.; ^2^ Department of Computer Engineering, University of West Attica, Athens, Greece.; ^3^ CREATE Team, XLIM Research Institute (UMR CNRS 7252), University of Limoges, Limoges, France.

## Abstract

As synthetic genomics scales toward the construction of increasingly larger genomes, computational strategies are needed to address technical feasibility. We introduce an algorithmic framework for the minimum-cost synthetic genome planning problem, aiming to identify the most cost-effective strategy to assemble a target genome from a source genome through a combination of reuse, synthesis, and join operations. By comparing dynamic programming and greedy heuristic strategies under diverse cost regimes, we demonstrate how algorithmic choices influence the cost efficiency of large-scale genome construction. In parallel, solving the minimum-cost synthetic genome planning problem can help us better understand genome architecture and evolution. Using both single closely related templates (e.g., bat coronavirus RaTG13) and diverse multisource consensus analyses, our results revealed that conserved regions such as ORF1ab can be reconstructed cost-effectively via sequence reuse. In contrast, highly variable regions such as the S (Spike) gene necessitate expensive *de novo* DNA synthesis. This highlights a concrete biological and economic trade-off in genome design: evolutionary sequence conservation dictates the financial feasibility of fragment reuse, whereas rapid viral adaptation incurs high synthesis penalties.

## Introduction

Synthetic genomics has expanded rapidly in recent years, offering unprecedented opportunities to understand genome function and engineer organisms with novel capabilities [1,2]. Major milestones include the synthesis of a functional poliovirus genome of approximately 7,500 base pairs (bp) in length [3], followed by the synthesis of the first self-replicating artificial cell, *Mycoplasma capricolum* [4], and the design and chemical synthesis of *Saccharomyces cerevisiae* chromosomes [5].

While DNA sequencing costs have plummeted precipitously, *de novo* DNA synthesis remains comparatively expensive and is strictly bottlenecked by sequence length and fidelity limitations [6,7]. Consequently, constructing large genomes currently requires synthesizing short fragments and hierarchically stitching them together via methods such as Golden Gate assembly, Gibson assembly, or *in vivo* DNA assembly [4,8,9]. To bypass these financial and technical restrictions, researchers can employ DNA reuse, extracting and amplifying existing, physically available DNA sequences. Integrating DNA reuse strategically minimizes the reliance on *de novo* synthesis, thereby drastically decreasing overall construction costs and improving the feasibility of assembling massive sequences.

To navigate these complexities, computational tools such as Raven [10] and DNALD [11] provide heuristic solutions for assembly planning. However, the fundamental problem definition and optimization objectives of these tools differ substantially from our approach. Existing software is primarily designed for the complex task of assembling combinatorial DNA libraries, where the goal is to maximize shared intermediate parts across many different target molecules to reduce parallel pipetting steps. Because minimizing shared parts across a library is an NP-hard problem [11], these tools rely on approximate heuristic solutions. In contrast, our framework focuses on the global, mathematical minimization of absolute financial cost for constructing a single, massive target genome.

Concurrently, foundational work in computational biology has focused on modeling the evolutionary distance between genomes. These models employ a set of “moves” intended to represent large-scale mutational events, including operations such as inversions, translocations, and the more general double cut and join [12–16]. However, these theoretical models are purely evolutionary distance metrics; they do not consider the asymmetric experimental cost structures, such as the severe price difference between synthesizing a novel sequence versus polymerase chain reaction (PCR)-amplifying an existing one, that govern actual laboratory work.

Here, we define and solve the minimum-cost synthetic genome planning problem from a theoretical standpoint. We introduce a novel algorithmic framework that moves beyond the process-based metrics of library assembly and the evolutionary models of genome rearrangement. Instead, we utilize a set of operations (reuse, synthesis, and join) that directly correspond to laboratory procedures and incorporate a weighted cost system reflecting their distinct expenses. Our goal is to determine the most cost-effective strategy to construct a target genome by optimally partitioning it into blocks that are either replicated from an existing source genome or synthesized *de novo*. This work bridges the gap between classic rearrangement theory and the practical demands of genomic engineering and provides a novel lens through which to measure evolutionary distance.

## Methods

### Formal problem definition

Let the source genome be represented as a string $G_{S}$ and the target genome as a string $G_{T}$, both composed of characters from the nucleotide alphabet $\sum =\left\{A,C,G,T\right\}$. Our objective is to construct $G_{T}$ by concatenating a sequence of nonoverlapping blocks, $b_{1},b_{2},\ldots ,b_{k}$, such that their concatenation equals $G_{T}$. We further impose a practical fragment-length constraint: each block must have a length of at most W, i.e., $1\leq \left|b_{i}\right|\leq W$. This user-specified maximum block length W applies uniformly to both *de novo* synthesis and replication (reuse) operations. Biologically, this constraint reflects the practical upper limits of standard laboratory protocols: *de novo* synthesis of excessively long fragments suffers from exponentially decreasing yields and higher error rates, while amplifying massive fragments via PCR is prone to polymerase slippage, secondary structure interference, and amplification failure.

### Genomic operations and cost model

We define a set of operations based on a workflow inspired by Golden Gate assembly [17], where multiple DNA fragments are joined in a one-pot reaction.

A reuse operation selects a block b that occurs as an exact substring in the source genome(s) $G_{S}$. Reuse incurs a fixed cost $C_{\text{reuse}}$ per reused block (independent of block length), capturing primer design and PCR reagents.

In practice, determining whether a candidate block b is reusable requires a fast substring existence query against the source genome $G_{S}$. We therefore build a Ferragina–Manzini index (FM-index) over $G_{S}$ once and use it to test each candidate block during planning. A block is treated as reusable if the FM-index reports at least one occurrence of b in $G_{S}$. Importantly, an FM-index supports existence queries in $O\left(\left|b\right|\right)$ time (i.e., linear in the block length), which is independent of $\left|G_{S}\right|$; since $\left|b\right|\leq W$, these queries are effectively constant time per candidate block for a fixed W. When the source database consists of multiple genomes (e.g., a panel of related viruses), these sequences are concatenated into a single string separated by a unique delimiter character (e.g., $) that does not appear in the standard nucleotide alphabet. A single generalized FM-index is then built over this concatenated database. This approach allows the algorithm to perform simultaneous existence queries across all available source material in a single operation, while the delimiter prevents false-positive substring matches that cross the artificial boundaries between concatenated genomes.

A synthesis operation involves the *de novo* chemical synthesis of a block $B_{k}$. The cost of this operation, $C_{\text{synth}}$, is a function of the block’s length w, reflecting that longer fragments are substantially more expensive to synthesize. We model this as a linear function $C_{\text{synth}}\left(w\right)=w\cdot c_{s}$, where $c_{s}$ is a constant representing the cost per base. For our larger-genome experiments (bacterial and eukaryotic), we employ a nonlinear synthesis cost model to capture the disproportionate difficulty, error rate, and expense of synthesizing excessively long DNA fragments. Specifically, this extended cost function is defined as $C_{\text{synth}}\left(\ell \right)=c_{q}\ell^{2}+c_{s}\ell$, where $c_{q}$ is a user-defined quadratic penalty constant and $c_{s}$ remains the per-base linear cost.

A join operation represents the cost of assembling 2 adjacent blocks. In a Golden Gate context, this involves designing compatible overhangs and performing a ligation reaction. We abstract this into a fixed cost, $C_{\text{join}}$, which is incurred for each junction created between fragments. A construction of k blocks will therefore require k−1 join operations.

### The minimum-cost objective problem

Given $G_{S}$ and $G_{T}$ and the cost functions $C_{\text{reuse}}$, $C_{\text{synth}}\left(L\right)$, and $C_{\text{join}}$, the problem is to find a partition of $G_{T}$ into blocks $b_{1},b_{2},\ldots ,b_{k}$ that minimizes the total construction cost, defined as

$$\text{TotalCost}=\sum_{i=1}^{k}\text{AcquisitionCost}\left(b_{i}\right)+\left(k-1\right)C_{\text{join}}$$ (1)

where the AcquisitionCost for each block is either $C_{\text{reuse}}$ or $C_{\text{synth}}\left(\left|b_{i}\right|\right)$.

### Algorithms

To aid interpretation of the cost model, we define the break-even length $W_{\mathrm{BE}}$, which represents the fragment length at which synthesis and reuse incur equal cost under the chosen parameters. While this quantity provides useful intuition for understanding when each operation is favored, it is not explicitly required by the dynamic programming (DP) algorithm, which directly evaluates both reuse and synthesis costs for every candidate block. Thus, the optimal solution is determined independently of $W_{\mathrm{BE}}$, ensuring that the algorithm remains unbiased with respect to operation choice.

To solve the minimum-cost synthetic genome planning problem, we developed and compared 3 distinct algorithmic strategies. The first is a DP approach that guarantees an optimal solution, while the other 2 are greedy heuristics designed to find fast, approximate solutions based on different local optimization criteria.

### DP algorithm for optimal planning

To find the provably optimal solution, we employ DP. Let T be the target genome of length N. We define an array, DP, where $\mathrm{DP}\left[i\right]$ stores the minimum possible cost to construct the prefix of the target genome of length i, i.e., $T\left[1..i\right]$. The goal is to compute $\mathrm{DP}\left[N\right]$.

The base case is $\mathrm{DP}\left[0\right]=0$, representing zero cost to construct an empty prefix. The recurrence relation to compute $\mathrm{DP}\left[i\right]$ for i>0 is defined as follows:

$$\begin{array}{c} \mathrm{DP}\left[i\right]=\min_{1\leq w\leq W}(DP\left[i-w\right]+\text{Cost}(\text{T}[i-w+1..i])+C_{\text{join}}) \end{array}$$ (2)

where W is the maximum allowed block length, a parameter that reflects the practical upper limit on the length of a DNA fragment that can be reliably synthesized or amplified. The term $\text{Cost}\left(T\left[i-w+1..i\right]\right)$ is the acquisition cost of a block of length w ending at position i. This cost is determined by querying an FM-index built over the source genome $G_{S}$. If the block $T\left[i-w+1..i\;\right]$ occurs as a substring of $G_{S}$, its cost is $C_{\text{reuse}}$; otherwise, its cost is $C_{\text{synth}}\left(w\right)$. Each such existence query takes $O\left(w\right)$ time, i.e., linear in the candidate block length and independent of $\left|G_{S}\right|$.

The $C_{\text{join}}$ cost is omitted for the very first block (when i−w=0). By iterating through all possible last blocks for each position i, this algorithm explores the entire solution space and guarantees that $\mathrm{DP}\left[N\right]$ holds the global minimum construction cost.

The complete procedure is formalized in Algorithm 1. During preprocessing, an FM-index F is built once over the source genome S. The FM-index is a compressed full-text index that supports substring existence queries: given a candidate block b of length ℓ, the function FM-Query(F,b) returns the number of occurrences of b in S in $O\left(\ell \right)$ time, i.e., linear in the block length and independent of the size of S. In the main loop, the algorithm iterates over every position i of the target genome and considers all possible last-block lengths ℓ from 1 to $\min\left(i,W\right)$. For each candidate block $b=T\left[i-\ell +1,\;..,\;i\right]$, the FM-index is queried: if the block occurs in S, it is marked for reuse at fixed cost $C_{\text{reuse}}$; otherwise, it must be synthesized *de novo* at a length-dependent cost $c_{s}\cdot \ell$. A join cost $C_{\text{join}}$ is added for every junction between consecutive blocks, except when the block is the first fragment of the construction. The recurrence $\mathrm{dp}\left[i\right]=\min_{\ell }\left(\mathrm{dp}\left[i-\ell \right]+C_{\mathrm{acq}}+C_{j}\right)$ guarantees that $\mathrm{dp}\left[n\right]$ holds the globally minimum construction cost after all positions have been processed. In the event of boundary cases, such as if a target genome requires a block b that is technically longer than the entire source genome $G_{S}$, the internal FM-Query function will natively return 0 occurrences. This safely and correctly forces the algorithm into the else branch, defaulting to the *de novo* synthesis cost.

**Figure:**
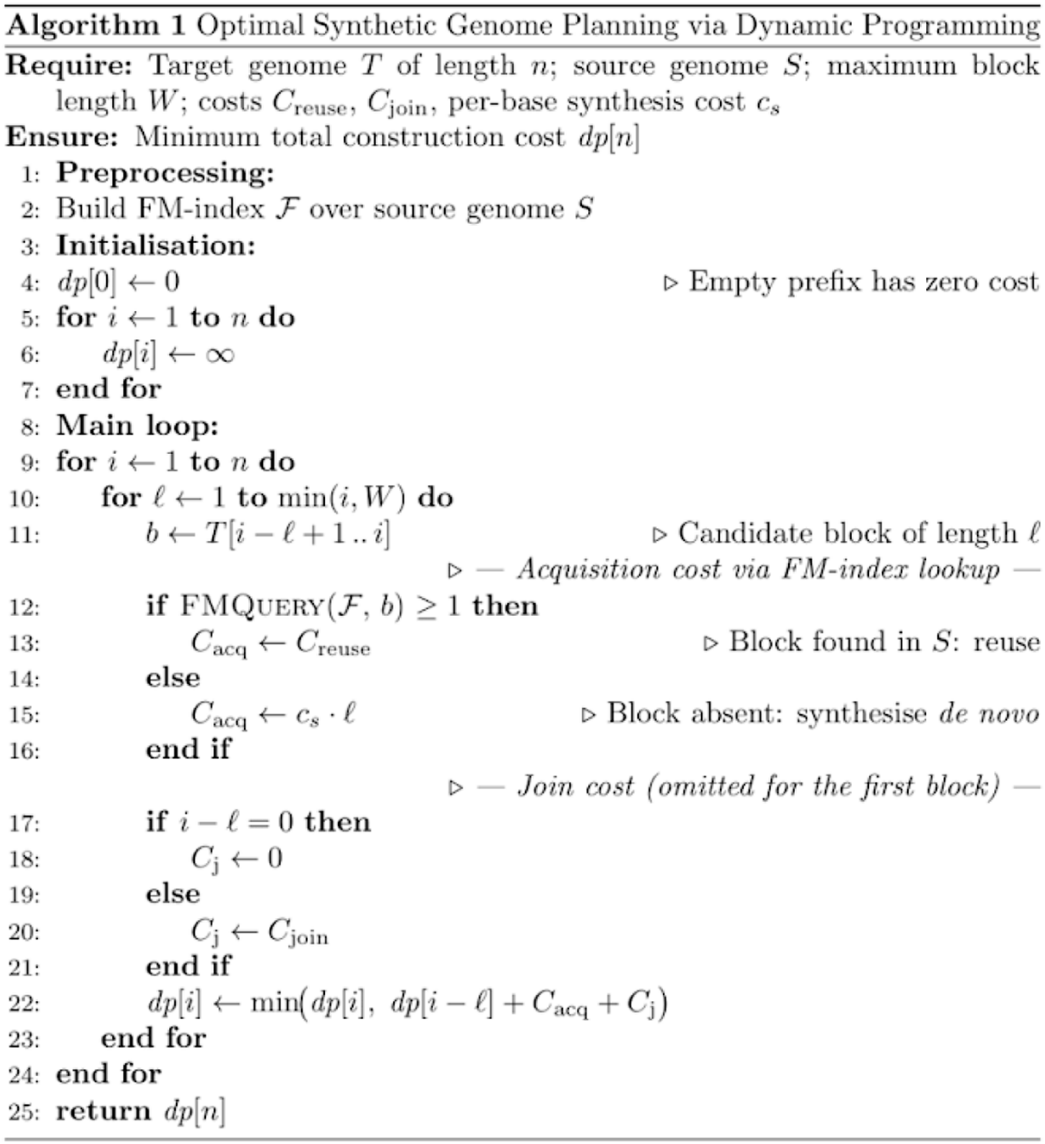

The optimality of the dynamic algorithm follows from the principle of optimal substructure. Any valid construction plan for the target genome $T\left[1..n\right]$ consists of a sequence of nonoverlapping blocks whose concatenation equals T. Consider an optimal plan whose last block has length ℓ and ends at position n. Removing this last block yields a plan for the prefix $T\left[1..n-\ell \right]$, and this subplan must itself be optimal: if a cheaper plan for $T\left[1..n-\ell \right]$ existed, substituting it in would reduce the total cost of the full plan, contradicting its optimality. The recurrence in Algorithm 1 explicitly enumerates every admissible last-block length $\ell \in \left[1,\;\min\left(i,W\right)\right]$ at every position i, and for each candidate, it selects the acquisition method (reuse or synthesis) that minimizes cost. Because the recurrence considers every possible partition boundary and every possible acquisition decision at each boundary, it explores the complete space of valid construction plans. By induction on i, $\mathrm{dp}\left[i\right]$ stores the minimum cost over all valid plans for the prefix $T\left[1..i\right]$. Consequently, $\mathrm{dp}\left[n\right]$ is the globally optimal construction cost for the entire target genome.

### Illustrative example

We illustrate the operations (reuse, synthesis, and join) and the resulting cost with a toy instance. Let the source genome be $G_{S}\;=\;\text{ACGTTGCA}$ and the target genome be $G_{T}\;=\;\text{ACGTTGCA}$, with a maximum allowed block length W=4. We use toy cost parameters: a fixed reuse (PCR) cost $C_{\text{reuse}}=5$, a per-base synthesis cost $c_{s}=2$ (so the synthesis model is $C_{\text{synth}}\left(\ell \right)=c_{s}\cdot \ell =2\ell$), and a join cost of $C_{\text{join}}=1$ per junction.

One feasible construction plan partitions the target into 3 blocks $B_{1}=\text{ACGT}$, $B_{2}=\mathrm{AA}$, and $B_{3}=\mathrm{GCA}$, so that $G_{T}=B_{1}\left\|B_{2}\right\|B_{3}$. Since $B_{1}=\text{ACGT}$ occurs as a substring of $G_{S}$, it can be acquired by reuse with a cost $\text{cost}\left(B_{1}\right)\text{=}\;C_{\text{reuse}}=5$; in contrast, $B_{2}=\mathrm{AA}$ does not occur in $G_{S}$ and must be synthesized, and because $\left|B_{2}\right|=2$, its cost is $\text{cost}\left(B_{2}\right)=C_{\text{synth}}\left(\left|B_{2}\right|\right)=2\cdot 2=4$. Finally, $B_{3}=\mathrm{GCA}$ again occurs in $G_{S}$ and is reused with a cost $\text{cost}\left(B_{3}\right)=C_{\text{reuse}}=5$. This plan uses m=3 blocks and therefore requires m−1=2 joins, contributing $2\cdot C_{\text{join}}=2\cdot 1=2$. The total construction cost of this plan is thus $\text{Total}=\left(5+4+5\right)+2\cdot 1=16$. This toy example makes explicit how conserved substrings in $G_{S}$ can be reused at a fixed cost, while novel segments in $G_{T}$ require length-dependent synthesis, and how increasing the number of blocks increases the join cost.

### Replication-first greedy algorithm

As a baseline for comparison, we implemented a greedy algorithm that prioritizes the reuse of existing genetic material. This “replication-first” strategy iterates through the target genome from start to finish. Starting at position i=0, it attempts to make a locally optimal choice by searching for the longest possible block starting at i (up to length W) that exists in the source genome S.

If such a replicable block of length w is found, the algorithm immediately selects it, adds $C_{\text{reuse}}$ and $C_{\text{join}}$ to the total cost (omitting $C_{\text{join}}$ if i=0), and advances the position by w. In practice, when the source genome contains the full nucleotide alphabet, a reusable short block is almost always available at each position. Thus, this heuristic typically behaves as a reuse-only baseline: it greedily selects the longest reusable block at each step, and differences in total cost are driven primarily by the number of fragments (and hence join operations). This process repeats until the entire target genome is constructed.

### Max-block greedy algorithm

We designed a second greedy heuristic to explore an alternative strategy focused on minimizing the number of join operations. This “max-block” algorithm always attempts to construct the target genome using the largest possible fragments. At each position i, it invariably considers the block of length $w=\min\left(W,\;N-i\right)$.

It then makes a local cost decision for this max-sized block. It first checks if the block exists in the source genome S. If it does, the algorithm chooses the cheaper option between replicating it (cost $C_{\text{reuse}}$) and synthesizing it (cost $C_{\text{synth}}\left(w\right)$). If the block does not exist in the source, the algorithm has no choice but to synthesize it at a cost $C_{\text{synth}}\left(w\right)$.

After selecting the block and adding the appropriate acquisition and join costs, the position is advanced by w. This strategy aggressively reduces the number of $C_{\text{join}}$ costs but may be forced into expensive synthesis operations that the replication-first or DP algorithms would avoid.

### Complexity analysis

#### Time and space complexity

Let N be the length (bp) of the target sequence to be assembled, W the maximum allowed block length, and $\left|G_{s}\right|$ the total length of the concatenated source genome database. The algorithm fundamentally consists of 2 phases: a one-time preprocessing phase and the DP optimization loop. During preprocessing, constructing the FM-index requires $O\left(\left|G_{s}\right|\right)$ time and space. During the optimization phase, the DP algorithm computes the cost array of length N, evaluating up to W candidate blocks at each position. Each candidate block requires an FM-index substring query, taking $O\;\left(W\right)$ time in the worst case. Therefore, the optimization loop requires $O\;\left(\mathrm{nW}\right)$ time and $O\;\left(n\right)$ space. Combining these phases, the total time complexity of our framework is strictly $O\left(\left|G_{s}\right|+\mathrm{nW}\right)$, and the total space complexity is $O\left(\left|G_{s}\right|+n\right)$. Because the FM-index construction scales linearly, i.e., $O\left(\left|G_{s}\right|\right)$ with the source database size, the overall theoretical complexity remains fundamentally linear with respect to the input sequence lengths. Explicitly including the $O\left(\left|G_{s}\right|\right)$ term is critical, however, as the computational and memory overhead of preprocessing becomes a dominant factor when evaluating massive reference databases (e.g., human-scale genomes or highly multiplexed multisource panels).

The problem formulated in this study belongs to complexity class P because the minimum-cost construction of a single, continuous target sequence exhibits a strict optimal substructure. Any optimal assembly plan for a sequence $T\left[1..i\right]$ inherently contains the optimal subplans for its prefixes. This sharply contrasts with related assembly planning problems, such as those addressed by DNALD [11], which aim to maximize shared intermediate parts across a combinatorial library of multiple targets simultaneously. Optimizing multitarget simultaneous assembly involves a set cover structure and is consequently NP-hard, necessitating heuristic approaches. By mathematically restricting our objective to the linear partition of a single target genome, our framework preserves the optimal substructure required to guarantee an exact, globally optimal solution in polynomial time via DP.

### DP solution

DP computes $\mathrm{DP}\left[i\right]$ for i=1,…,N and tries all last-block lengths $w\in \left\{1,\ldots ,W\right\}$ at each position. Therefore, it performs $\Theta \left(\mathrm{NW}\right)$ candidate transitions and FM-index queries, giving worst-case time $O\;\left(\mathrm{NW}\cdot W\right)=O\left(NW^{2}\right)$ if we account for the FM-index query cost $O\;\left(w\right)$, or $O\;\left(\mathrm{NW}\right)$ if FM-index queries are treated as $O\;\left(1\right)$ for a fixedW. DP uses $O\;\left(N\right)$ memory for the cost table (plus FM-index storage).

### Greedy replication-first

At each step starting at position i, the greedy searches for the longest reusable block length up to W. In the worst case, this checks up to W candidate lengths per step; over $O\;\left(N\right)$ steps, worst-case time is $O\left(NW^{2}\right)$ FM-index queries (or $O\;\left(\mathrm{NW}\right)$ if counting $O\;\left(1\right)$ per query). In typical genomes, the algorithm advances by long blocks, so the number of steps (and thus queries) is much smaller in practice.

### Greedy max-block

This heuristic always attempts the length-W block at the current position and then advances by W bases. It therefore makes about $\Theta \left(N/W\right)$ FM-index queries and runs in $O\;\left(\left(N/W\right)\cdot W\right)=O\left(N\right)$ time when counting FM-index query cost $O\;\left(W\right)$. It is typically the fastest but has the weakest optimality properties.

### Complexity class

We address whether the minimum-cost synthetic genome planning problem studied in this article is NP-hard or belongs to P. In our formulation, there is a source genome string S, a single target genome string T of length N, a maximum allowed block length W, and costs $C_{\text{reuse}}$ (reuse/PCR), $C_{\text{join}}$ (join), and $C_{\text{synth}}\left(w\right)$. A plan is a partition of T into blocks of length at most W, where each block is either reused if it occurs as a substring of S or synthesized otherwise.

As described in the “Time and space complexity” section, this formulation admits an exact DP algorithm that runs in time polynomial in N and W. Since a polynomial-time algorithm exists for this formulation, the corresponding decision version (“is there a plan of cost at most B?”) belongs to P, and so does the optimization problem.

### Computational performance and resource usage

All experiments were executed on an x86-based Slurm cluster with 16 central processing unit (CPU) cores allocated per run. For the viral pipelines, we report end-to-end wall-clock runtime and peak memory as recorded by the scheduler for the batch step. Under these conditions, the complete pipelines finish within seconds to a couple of minutes (approximately 3.6 to 101.4 s across S2 to S1) while maintaining a small memory footprint on the order of tens of mebibytes at peak.

For the larger-genome bacterial and eukaryotic analyses, we benchmarked the planner stage separately to isolate the computational cost of the optimization algorithm from one-time preprocessing (FM-index construction) and downstream plotting. Using 16 x86 CPU cores, the planner stage completes in 13.1 min for the bacterial sweep and 30.1 min for the eukaryotic sweep, with peak resident memory below 200 MiB in both cases (Table 1).

**Table 1.** FM-index construction time and load-time memory for representative viral, bacterial, and eukaryotic reference genomes. Build times are from the Slurm job logs produced by the provided build scripts; load time and peak RAM are measured by a small loader program that loads the serialized Succinct Data Structure Library (SDSL) FM-index (.fm) into memory and reports wall-clock time and peak resident set size (RSS) during loading.

Genome class	Assembly (accession)	Build time (s)	Load time (s)	Peak RSS on load (MiB)	Index size in memory (MiB)
Viral	SARS-CoV-2 reference (NC_045512.2)	0.149	0.00371	2.813	0.015
Bacterial	*Escherichia coli* K-12 MG1655 (NC_000913.3)	1.180	0.0200	2.813	1.643
Eukaryotic	Human GRCh38.p14 (GCF_000001405.40)	819.658	1.370	1,632.000	1,631.810

FM-index, Ferragina–Manzini index; RAM, random-access memory; SARS-CoV-2, severe acute respiratory syndrome coronavirus 2

The overall workflow is readily scalable: preprocessing is performed once per source genome, and planner executions are independent across sources and can therefore be distributed across nodes (e.g., via job arrays) to increase throughput. This makes it practical to extend the analysis to larger panels of source genomes and to larger targets while keeping per-task memory requirements modest and enabling near-linear scaling in aggregate runtime with available compute resources.

### The break-even length

To rigorously evaluate our algorithms under diverse economic trade-offs, we introduced a unified metric called the break-even length, denoted as $W_{\mathrm{BE}}$. This value represents the crossover block length below which *de novo* synthesis is cheaper than replication and above which replication becomes the more cost-effective option. Specifically, for any block shorter than $W_{\mathrm{BE}}$, the linear synthesis cost $\left(c_{s}\cdot l\right)$ is less than the fixed reuse cost, making synthesis preferred; for blocks longer than $W_{\mathrm{BE}}$, the opposite holds and replication is favored. This trade-off is captured by the inequality $C_{\text{synth}}\left(w\right)\leq C_{\text{reuse}}$.

Given our linear cost model where $C_{\text{synth}}\left(w\right)=w\cdot c_{s}$, this inequality becomes $w\cdot c_{s}\leq C_{\text{reuse}}$. By solving for w, we can identify the range of lengths where synthesis is the cheaper acquisition method. The break-even point occurs at $W_{\mathrm{BE}}=C_{\text{reuse}}/c_{s}$. For any block shorter than $W_{\mathrm{BE}}$, synthesis is preferred, while for any block longer than $W_{\mathrm{BE}}$, replication is favored. By systematically varying the $W_{\mathrm{BE}}$ value (by adjusting $C_{\text{reuse}}$ and $c_{s}$ while holding $C_{\text{join}}$ constant), we can efficiently explore the entire spectrum of economic pressures and test the robustness of each algorithm’s decision-making strategy.

It is important to note that this specific algebraic derivation $\left(W_{\mathrm{BE}}=C_{\text{reuse}}/c_{s}\right)$ applies strictly to the linear synthesis cost model utilized in our viral analyses. In subsequent experiments involving larger genomes, we introduce a more realistic nonlinear synthesis model containing a quadratic penalty term. Under these conditions, the theoretical break-even length shifts and must be solved as the positive root of the quadratic equation $c_{q}\ell^{2}+c_{s}\ell -C_{\text{reuse}}=0$.

### Genomic analysis of viral genomes

To demonstrate the utility of our framework, we performed 3 computational case studies using viral genomes. These analyses were designed to (a) empirically evaluate the performance of the greedy heuristics against the optimal DP algorithm, (b) quantify the relationship between source–target genome similarity and construction cost, and (c) investigate whether cost profiles can reveal features of genome architecture. Throughout the paper, we use performance to refer to solution quality, measured by the construction cost returned by an algorithm (lower is better).

#### Analysis 1: Algorithm cost performance comparison

To conduct a robust comparison of the DP, replication-first greedy, and max-block greedy algorithms, we performed a leave-one-out assembly simulation using a diverse set of 84 viral genomes (Table S1). Unlike predictive machine learning models, where sequence homology causes data leakage, our deterministic planner explicitly relies on the presence of shared genetic material. Including closely related strains realistically mimics a laboratory freezer inventory. For each of the 84 viruses in the dataset, it was designated as the target genome. The corresponding source genome was constructed by concatenating the remaining 83 viruses, from which a single FM-index was built. We then calculated the construction cost for each of the 3 planners. This entire process was repeated for a range of 8 distinct “break-even length” $\left(W_{\mathrm{BE}}\right)$ values, from 5 to 25, to assess performance across different economic trade-offs between replication and synthesis.

#### Analysis 2: Impact of source genome similarity on construction cost

To investigate how evolutionary distance impacts economic feasibility, we calculated the optimal construction cost of a fixed target genome from a panel of source genomes with varying degrees of similarity. The reference genome of severe acute respiratory syndrome coronavirus 2 (SARS-CoV-2; NC045512.2) was selected as the target. A curated set of 12 source genomes from the *Coronaviridae* family was used, chosen to provide a range of genetic similarities (Table S2). For each of the 12 source genomes, a dedicated FM-index was built. The optimal construction cost of the target was then calculated using this index. This procedure was repeated for 3 distinct economic scenarios: “replication-dominant”, “high-join-cost”, and “balanced”. The similarity between each source and the target was quantified post hoc using the average nucleotide identity (ANI), estimated with the MASH software.

#### Analysis 3: Genome architecture cost profiling

To test the hypothesis that our cost model can identify functionally conserved and variable genomic regions, we generated a “cost profile” of the SARS-CoV-2 genome (NC045512.2). This was accomplished by modifying our DP planner to output the entire cost array, $\mathrm{DP}\left[0\ldots N\right]$. The local construction cost for a given region was then calculated using a 500-bp sliding window. This analysis was performed in 2 distinct experiments. First, a single cost profile was generated using the closely related bat coronavirus (CoV) RaTG13 (MN996532.2) as the source. Second, to obtain a consensus view, cost profiles were generated from a curated set of 8 different CoV source genomes (Table S3). These individual profiles were then used to calculate a mean cost profile and its standard deviation across the target genome. Both experiments were run using the “balanced” cost parameters ($C_{\text{reuse}}=5$, $C_{\text{join}}=1.5$, and $C_{\text{synth}}=0.2$) with a maximum block length W=100.

## Results

### Analysis of costs across the algorithms

**Fig. 1.**
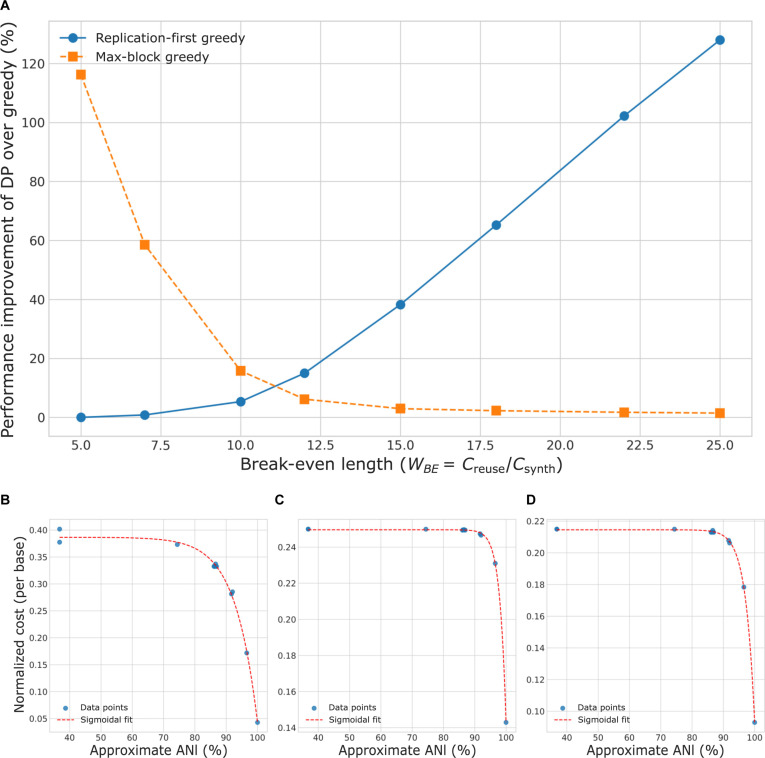
Total generation cost *vs.* source genome similarity. (A) Difference in mean normalized total cost between each greedy heuristic and the optimal dynamic programming (DP) planner as a function of the break-even length $W_{\mathrm{BE}}$. The *y*-axis reports the absolute normalized cost gap, defined as $\left(C_{\text{greedy}-C_{\mathrm{DP}}}\right)/L$, where L is the target genome length. Values are averaged over all target genomes; positive values indicate that greedy heuristics are more expensive than the DP optimal. (B to D) The relationship between the optimal construction cost and the source genome similarity (average nucleotide identity [ANI] %) under 3 distinct cost regimes: (B) replication-dominant, (C) high-join-cost, and (D) balanced.

We compared the performance of the 2 greedy heuristics relative to that of the optimal DP planner. The performance penalty is reported as the normalized cost gap—defined as the absolute difference in construction cost per base pair between the greedy heuristic and the optimal DP solution. The results demonstrate that while the DP algorithm consistently finds the optimal solution, the performance of the greedy algorithms is highly sensitive to the economic conditions defined by the break-even length ($W_{\mathrm{BE}}$) (Fig. 1A). The replication-first greedy algorithm, which is designed to prioritize the reuse of genetic material, performs well when $W_{\mathrm{BE}}$ is low, as this corresponds to scenarios where replication is almost always the correct and cheapest choice. However, as $W_{\mathrm{BE}}$ increases, the performance gap grows to over 200%. This is because a high $W_{\mathrm{BE}}$ value creates a wide range of *k*-mer lengths for which synthesis is the cheaper option. The greedy algorithm falls into an “economic trap” by repeatedly choosing to pay the high fixed cost of replication ($C_{\text{reus}e}$) for short *k*-mers, while the DP planner correctly identifies that synthesizing these fragments is the more cost-effective global strategy.

Conversely, the max-block greedy algorithm, designed to minimize join operations by always selecting the largest possible *k*-mer, exhibits the opposite behavior (Fig. 1A). However, as the break-even length approaches or exceeds the maximum block length constraint ($W_{\mathrm{BE}}\geq W$), the cost of synthesis becomes universally cheaper than the fixed cost of replication for all valid fragment lengths. Under these specific economic conditions, the theoretically optimal global strategy is simply to minimize the number of join operations by partitioning the genome entirely into synthesized fragments of maximum length W. Because the max-block strategy is explicitly designed to always select W-length fragments, its behavior perfectly aligns with the DP algorithm’s optimal strategy in this regime. Consequently, its performance gap mathematically drops to zero, entirely independent of the sequence similarity between the source and target genomes.

### Impact of source genome similarity on construction cost

To confirm the relationship between the optimal construction cost and the evolutionary distance of the source genome, we conducted a computational experiment using the SARS-CoV-2 reference genome as a fixed target. A curated set of source genomes was selected, primarily from the *Coronaviridae* family, to provide a range of genetic similarities. The similarity between each source and the target was quantified using the ANI, a robust measure of genome-wide sequence identity, which we estimated using the MinHash algorithm implemented in the MASH software [18]. We then calculated the minimum construction cost under 3 distinct economic scenarios: “replication-dominant”, “high-join-cost”, and “balanced”.

The results demonstrate a strong negative correlation between the construction cost and the ANI of the source genome across all tested scenarios (Fig. 1B to D). As the source genome becomes more genetically similar to the target, the normalized cost per base decreases dramatically. This trend is most pronounced in the “replication-dominant” scenario, where the cost plummets by over 85% as the ANI increases from 36.79% to 100%. This occurs because higher similarity allows the DP planner to utilize a greater number of large, cost-effective $C_{\text{reuse}}$ operations, substantially reducing the reliance on expensive $C_{\text{synth}}$ operations. To establish a true minimum-similarity boundary, non-CoV outgroups (enterobacteria phages EcoDS1 and phiX174) were intentionally included, resulting in the sparse data points below 80% ANI (e.g., enterobacteria phage EcoDS1 at 36.79% ANI). These outgroups serve as a strict baseline control where no appreciable *k*-mer reuse is biologically possible, causing the algorithm’s cost to converge to a maximum determined almost entirely by *de novo* synthesis—effectively representing the economic ceiling of building the target genome entirely from scratch. These results are consistent with the expectation that a cost-minimization framework of this kind would capitalize on genetic conservation and that the economic feasibility of synthetic genome construction is linked to the presence of a closely related template organism.

### Cost signatures distinguish conserved and rapidly evolving genomic regions

**Fig. 2.**
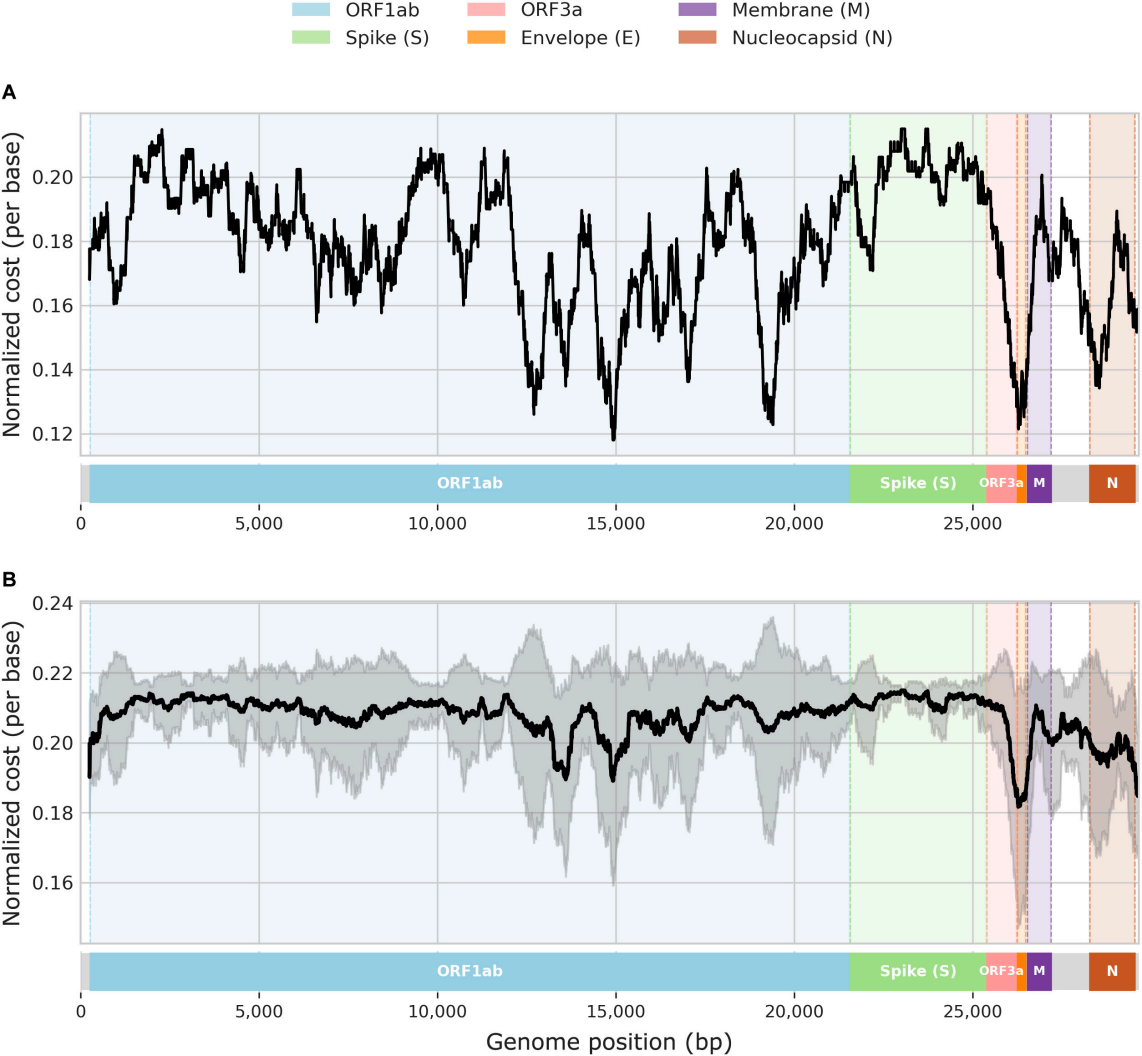
Genome architecture cost profile of severe acute respiratory syndrome coronavirus 2 (SARS-CoV-2). The optimal construction cost to build SARS-CoV-2 from (A) a closely related source (bat coronavirus [CoV] RaTG13) and (B) the mean cost from 8 different coronavirus sources. The genomic track bar beneath each plot delineates the boundaries of major reading frames, highlighting the corresponding regional cost spikes (e.g., the Spike [S] gene) and troughs (e.g., ORF1ab).

To further confirm the relationship between our economic cost model and the functional architecture of the genome, we generated a “cost profile” by calculating the optimal construction cost over a sliding window for the SARS-CoV-2 target genome (Fig. 2). This analysis was performed first using a single, closely related source genome (bat CoV RaTG13) and subsequently averaged across multiple diverse CoV sources to obtain a consensus view.

The results, summarized in Table 2, reveal a distinct cost signature that correlates with known biological functions. In both the single-source and multisource analyses, the Spike (S) gene region consistently incurs the highest mean construction cost. This is consistent with the known biology of the Spike protein, which is a surface glycoprotein responsible for host cell receptor binding and is known to be a primary target of the host immune system. Consequently, it is under intense selective pressure to mutate, leading to high sequence divergence even among closely related viruses. Our framework captures this evolutionary volatility as a high economic cost, as the substantial number of differing base pairs necessitates more frequent and expensive *de novo* synthesis operations.

**Table 2.** Combined mean cost analysis per gene region

Gene region	Single profile	Grouped analysis	
	Mean cost	Mean cost	SD
ORF1ab	0.1705	0.2082	0.0133
Spike (S)	0.1998	0.2125	0.0055
ORF3a	0.1680	0.2053	0.0163
Envelope (E)	0.1297	0.1835	0.0323
Membrane (M)	0.1752	0.2036	0.0160
Nucleocapsid (N)	0.1599	0.1975	0.0192

Conversely, the large ORF1ab region exhibits a markedly lower construction cost in the single-source comparison. As expected, while the Spike region has the highest average cost in the multisource analysis, its standard deviation is comparatively low. It is important to note that this standard deviation represents the variance in construction cost across the 8 diverse CoV source genomes used in the panel; the single-source analysis inherently lacks a standard deviation as it is a deterministic calculation from one template. The low variance for the Spike gene suggests that its high degree of divergence from SARS-CoV-2 is a consistent, universal feature across the broader CoV family. In contrast, other accessory genes like the Envelope (E) show a higher variance, indicating more diverse, source-dependent evolutionary patterns within the group.

### Scaling to larger bacterial and eukaryotic genomes

**Fig. 3.**
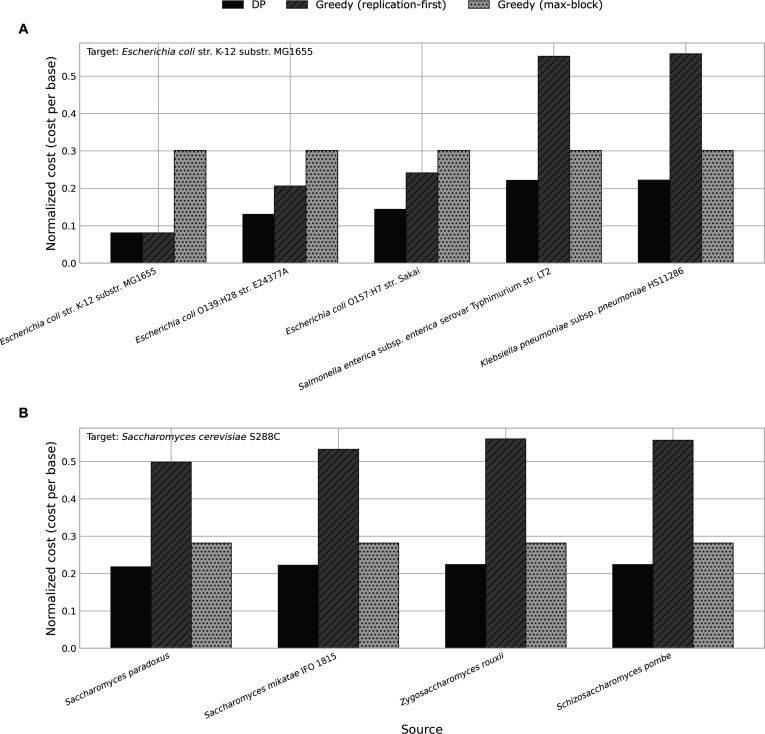
Normalized construction cost when constructing a fixed target genome from a single source genome; source genomes are ordered from more similar to less similar to the target (left to right). (A) Bacterial (target: *Escherichia coli* K-12 MG1655; parameters: W=1,000, $C_{\text{reuse}}=5$, $C_{\text{join}}=1.5$, $C_{\text{synth}}=0.2$, and $C_{\text{synth}2}=10^{-4}$). (B) Eukaryotic (target: *Saccharomyces cerevisiae* S288C; parameters: W=800, $C_{\text{reuse}}=5$, $C_{\text{join}}=1.5$, $C_{\text{synth}}=0.2$, and $C_{\text{synth}2}=10^{-4}$).

To evaluate whether the cost-minimization framework extends beyond viral targets, we performed 2 additional sweeps on substantially larger genomes (Table S4): a bacterial target (*Escherichia coli* K-12 MG1655) and a eukaryotic target (*S. cerevisiae* S288C). In each sweep, the target genome is fixed and we construct it from a single source genome chosen to span a range of similarity to the target (ordered from more similar to less similar, left to right in Fig. 3, as quantified by MASH-estimated ANI). This experimental design mirrors the viral similarity analysis, but at a larger genomic scale.

In these larger-genome experiments, synthesis was modeled using a nonlinear cost that includes a quadratic term, so that synthesizing a segment of length ℓ contributes $C_{\text{synth}}\cdot \ell +C_{\text{synth}2}\cdot \ell^{2}$ in addition to reuse and join costs. Relative to a purely linear synthesis model, this convex penalty discourages excessively long synthesized segments and provides a more realistic trade-off between reusing available blocks and synthesizing new material when planning large constructs.

Figure 3 summarizes the resulting normalized costs (cost per base) for each algorithm across the ordered sources. In the bacterial sweep (Fig. 3A), the overall construction cost increases as sources become less similar to the fixed target, consistent with the reduced availability of long exact reusable blocks. In contrast, the eukaryotic sweep (Fig. 3B) shows only modest variation across the selected sources, indicating that under exact-match reuse and the chosen economic parameters, the optimal plan is comparatively insensitive to which nonidentical yeast source is used. Crucially, the successful implementation of this nonlinear cost model for larger genomes highlights the algorithmic generalizability of our framework. While a simple linear model suffices for short viral fragments, the DP planner seamlessly adapts to optimize under the more complex quadratic synthesis constraints required for bacterial and eukaryotic targets. Across both linear and nonlinear cost regimes, the DP planner remains the robust, lowest-cost method relative to the greedy baselines, underscoring the necessity of global optimization when reuse, joins, and synthesis compete.

## Discussion

Our goal in this work was to model genome construction strategies that balance *de novo* DNA synthesis with the reuse of existing DNA fragments. The strong correlation between construction cost and source–target genome similarity highlights that economic feasibility is tightly linked to evolutionary conservation. While the cost parameters utilized in our simulations (e.g., $C_{\text{reuse}}$ and $c_{s}$) were standardized to facilitate clear algorithmic comparisons, real-world genome synthesis costs are highly dynamic [19]. Vendor pricing, economies of scale, and institutional reagent discounts fluctuate. Our framework is explicitly parameterized to accept these variables as dynamic inputs, ensuring that the planner remains practically relevant and capable of generating optimal strategies tailored to a specific laboratory’s exact budgetary reality.

To translate our abstract mathematical model into a direct laboratory tool, future iterations must embed specific design rule constraints directly into the DP recurrence. Currently, our framework abstracts the assembly process by applying a uniform, sequence-independent penalty ($C_{\text{join}}$) to every newly created junction. In physical practice, however, established assembly protocols such as Golden Gate [17] and Gibson assembly [20] impose strict sequence constraints. This can be computationally achieved through 2 mechanisms. First, hard biochemical or commercial limitations can be implemented by pre-evaluating the sequence of a candidate block. For example, commercial vendors routinely reject *de novo* synthesis orders for fragments containing severe tandem repeats, homopolymers, or extreme GC content [21]. Similarly, highly multiplexed Golden Gate assembly requires the design of unique, orthogonal 4-bp overhangs at every junction, and any reusable block containing internal type IIS restriction sites (e.g., *Bsa*I) will disrupt the assembly. If a target block violates these rules, the algorithm can assign it an acquisition cost of infinity (∞), strictly pruning it from the DP search space. Second, while our current model treats the DNA reuse (PCR) cost as a fixed constant dominated by primer pricing, amplifying exceptionally large fragments often requires expensive specialized polymerases. The DP recurrence can trivially accommodate this by evolving the scalar $C_{\text{reuse}}$ into a length-dependent function $C_{\text{reuse}}\left(\ell \right)$. Furthermore, soft thermodynamic constraints can be integrated by replacing the static join cost constant with a sequence-dependent penalty function that penalizes junctions with extreme GC/AT content or poor orthogonality scores.

A natural extension of the current framework is to relax the exact-match requirement to better reflect the flexibility of wet-lab protocols, specifically regarding site-directed mutagenesis. Currently, our zero-tolerance exact matching dictates that even a single-nucleotide polymorphism forces the algorithm to classify the entire segment as requiring *de novo* synthesis. Economically, incorporating mismatch tolerance introduces a new acquisition operation: mutagenesis. This operation would carry a fixed cost marginally higher than exact reuse due to specialized primer design but substantially lower than length-dependent synthesis. Theoretically, allowing a Hamming distance of k≤2 per block would rescue large fragments from full synthesis, effectively restoring the low-cost “replication-dominant” regime despite the presence of synonymous mutations. To quantitatively estimate how often this exact-match constraint is binding, we can evaluate expected block availability in our viral case studies under a uniform mutation assumption. At 99% ANI—representing closely related strains—a target sequence differs from the source by roughly one point mutation every 100 bp. Under our strict exact-match requirement, the probability of successfully reusing a maximum-length block of W=200bp is mathematically constrained to ${\left(0.99\right)}^{200}\approx 13.4\%$. Consequently, over 86% of potential 200-bp fragments are strictly bound by this constraint and forced into the costly *de novo* synthesis pipeline. If a tolerance of up to 2 mismatches were permitted via PCR with mismatch primers, the cumulative binomial probability of successfully finding a reusable 200-bp block would rise to approximately 67%. We estimate that this relaxation would reduce total modeled construction costs by an additional 30% to 50% for highly similar viral strains. This quantitative impact underscores the limitation of exact-match boundaries. While an exact query for a block of length ℓ runs in $O\left(\ell \right)$ time, the standard FM-index backtracking approach for approximate matching with up to k mismatches expands the search tree exponentially. However, modern algorithms can severely curtail this worst-case overhead through aggressive boundary pruning [22]. Furthermore, adopting bidirectional FM-index search schemes or seed-and-extend heuristics can effectively prune the search space. We are currently working on developing a more optimal solution utilizing these techniques, which will tolerate up to k mismatches to fully capture the economics of site-directed mutagenesis in future iterations.

While our DP framework identifies the mathematically absolute cost minimum, transitioning this abstract plan to the physical bench requires secondary validation. For example, processing a DP-derived assembly plan for SARS-CoV-2 through standard wet-lab software, such as the NEB Golden Gate Assembly Tool, would likely reveal specific junctions requiring minor positional shifts to achieve optimal thermodynamic overhang stability or to eliminate unintended internal restriction sites. Future software iterations will aim to integrate these secondary feasibility checks natively.

Our analysis revealed that the eukaryotic target (*S. cerevisiae*) exhibited unexpectedly low sensitivity to the similarity of the source genome compared to the viral and bacterial datasets. This effect likely reflects the limited availability of long, exact reusable segments between nonidentical yeast genomes. Because the probability of exact *k*-mer matches drops precipitously as the sequence length increases, the planner is forced to rely more heavily on *de novo* synthesis. Under our nonlinear cost model, these synthesis costs dominate the total expense, effectively washing out the minor savings gained from short fragment reuse.

Furthermore, our current framework abstracts the physical joining process through a fixed cost ($C_{\text{join}}$) applied to adjacent fragments. While this accurately models single-pot reactions for smaller genomes, it does not fully capture the complexities of constructing genomes exceeding 100 kb. At that scale, stepwise, hierarchical assembly (e.g., using bacterial or yeast artificial chromosomes) becomes a necessity. These multistage build strategies incur additional overhead for intermediate assemblies, transformation, and validation. Integrating hierarchical assembly cost models, where intermediate fragments are grouped into subassemblies with their own discrete processing costs, represents an important future extension of this framework to fully encompass genome-scale synthesis [4,5].

Crucially, the mathematical optimality of our DP framework is perfectly preserved in multisource genome scenarios. Because the FM-index inherently supports generalized indexing by concatenating multiple reference sequences (separated by unique delimiter characters), the planner treats a massive multisource database, such as the 84-virus panel used in our assembly simulations, as a unified, contiguous search space. Consequently, the algorithm evaluates all available source templates simultaneously, guaranteeing that the final construction plan is the global minimum cost across the entire provided inventory. The primary limitation to scalability in this multisource context is not algorithmic complexity, but rather the system random-access memory (RAM) required to hold the generalized FM-index, which scales linearly with the total aggregate size of the source database.

## Data Availability

All source code, analysis scripts, and data required to reproduce the findings of this study are open-source and publicly available in 2 separate repositories. The standalone C++ implementation of the DP algorithm is available at GenomePlanner: https://github.com/Georgakopoulos-Soares-lab/GenomePlanner. This command-line tool is designed for efficiency, leveraging the sdsl-lite library [23] to construct an in-memory FM-index of the source genome for rapid substring queries. The tool takes as input a source and a target genome in FASTA format and the key cost parameters from our model: --W for maximum block length (W), --pcr for the reuse cost (C_reuse), --join for the join cost (C_join), and --synth for the per-base synthesis cost (c_s). To ensure the full reproducibility of our findings, all analysis pipelines, processing scripts, and configuration files used to generate the figures and results in this study are available at Minimum_cost_Genome_Planner: https://github.com/Georgakopoulos-Soares-lab/Minimum_cost_Genome_Planner.
